# A Rare Coincidence of Two Coronary Anomalies in an Adult

**DOI:** 10.4061/2010/376067

**Published:** 2010-05-16

**Authors:** Cesar Cruz, Dalton Mclean, Matthew Janik, Paolo Raggi, A. Maziar Zafari

**Affiliations:** ^1^Division of Cardiology, Atlanta Veterans Affairs Medical Center, Decatur, GA 30033-4004, USA; ^2^Division of Cardiology, Emory University, Atlanta, GA 30322, USA

## Abstract

Anomalous right-sided left main coronary arteries and dual type IV left anterior descending arteries are rare coronary anomalies. In this case report, we present a 59 year old man with atypical chest pain and a combination of the above coronary anomalies as identified by selective coronary angiography and computed tomography angiography. To the best of our knowledge, the coincidence of these coronary anomalies has not been previously described.

## 1. Introduction

Anomalies of the coronary arteries occur in 0.2%–1.2% of the general population [1–3]. Anomalous left main coronary artery (LMCA) arising from the right sinus of Valsalva is found in 0.0024% to 0.03% of the population [2, 4]. Dual left anterior descending coronary artery (LADCA) is a rare congenital anomaly seen in about 1% of the population and is classified in four different types [5]. It is important to recognize both coronary anomalies due to different clinical implications. 

In this case report, we present a patient with an anomalous LMCA arising from the right sinus of Valsalva that divides into a left circumflex coronary artery (LCXCA) and a long LADCA and an additional hypoplastic LADCA arising from the left sinus of Valsalva.

## 2. Case Report

**Figure 1:**
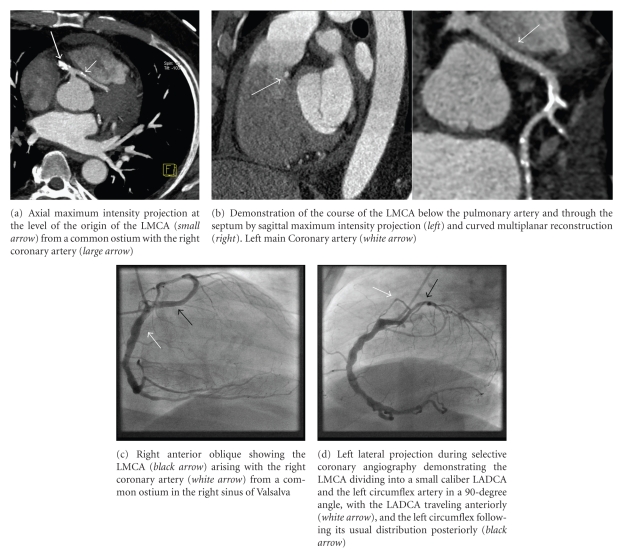
Transseptal course of the right-sided left main coronary artery by CTA and selective coronary angiography.

**Figure 2:**
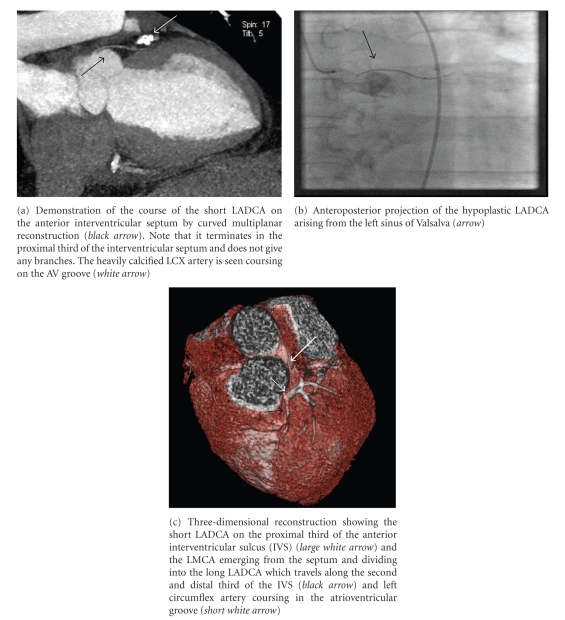
Short LADCA (hypoplastic) arising from the left sinus of Valsalva by CTA and selective coronary angiography.

 A 59-year-old man with a history of type 2 diabetes mellitus, hypertension, and dyslipidemia was referred to cardiology clinic for evaluation of atypical chest pain. The patient denied any family history of coronary artery disease and had a history of 60 pack years of smoking. Physical examination was normal as was the 12-lead electrocardiogram. He had a recent workup, including transthoracic echocardiography that showed a left ventricular ejection fraction of 60%, stage II diastolic dysfunction and mild biatrial enlargement, and a radionuclear myocardial perfusion imaging stress test that showed no evidence of inducible ischemia or previous myocardial infarction. Patient underwent cardiac catheterization for definite evaluation of coronary artery disease. Selective right coronary angiography with a 6 F right coronary Judkins 4 catheter revealed a common blood vessel from which the right coronary artery (RCA) and the LMCA were arising. The RCA followed its normal course around the atrioventricular groove and the LMCA traveled to the left side of the heart and divided at the level of the interventricular septum giving off a long LADCA and the LCXCA (Figure 1). Selective left coronary angiography with a 6F left Judkins 3.5 catheter revealed a second short hypoplastic LADCA that ended at the distal end of the proximal third of the interventricular sulcus without giving any branches of significant size (Figure 2). An aortogram was done to demonstrate the origins of the coronary blood vessels. Four coronary arteries were identified, one arising from the left coronary sinus and the other three originating from a main blood vessel from the right sinus of Valsalva. Left ventriculography showed normal chamber size and wall motion. There was significant calcification of the proximal segments of the RCA, LMCA, the longer LADCA, and the LCXCA without significant diameter stenoses. A coronary computed tomography angiography (CTA) was ordered to further delineate the course of the longer LADCA. Images were acquired with prospective gating on a 64-Multislice detector computed tomography scanner (Somatom Definition, Siemens, Forchheim, Germany) in the craniocaudal direction during suspended respiration at 0.75 mm slice thickness and reconstruction interval, 0.33 second gantry rotation speed, tube voltage 120 kVp, and a peak tube current of 390 mA. Cumulative dose-length product was 651 mGy × cm. Iso-osmolar nonionic contrast material (Omnipaque, GE Healthcare, Princeton, NJ) was used. Premedication with nitroglycerin 0.6 mg SL was administered. No beta blocker was given since heart rate was <70 per minute. CTA demonstrated the LMCA traveling to the left side of the heart behind the right ventricular outflow tract and through the interventricular septum (Figures 1 and 2). No angiographically significant stenoses or high-degree calcifications were noted.

## 3. Discussion

Coronary anomalies are frequently asymptomatic and usually discovered as incidental findings during diagnostic cardiac catheterization [6]. Its recognition may have significant clinical implications. 

Spindola-Franco et al. classified dual LAD coronary arteries into four types. Types I to III consist of a LADCA that divides into a shorter and a longer LADCA. These arteries are further differentiated by the course that the longer LADCA takes in relation to the interventricular septum in order to reach the apex. Type IV consists of a shorter LADCA originating in the left sinus of Valsalva and a longer LADCA arising from the right coronary artery [5]. In a recent large series involving 70,850 unselected patients, a Type IV LAD was noted only in 3 out of 171 (1.8%) of patients with major congenital coronary anomalies [7]. Our case patient is most consistent with a dual LADCA Type IV, as a longer LADCA arises from the anomalous right-sided LMCA, and a shorter LADCA emerges from the left coronary artery sinus and travels along the anterior proximal third of the interventricular septal wall. 

Routine coronary angiography may fail to demonstrate the longer LADCA, particularly if it arises from a separate ostium, and could lead to erroneous interpretation of a 100% mid-LADCA occlusion. In such cases, aortography in the left anterior oblique projection may help to visualize the origin of this anomalous blood vessel. In our case, the short hypoplastic LADCA supplies a very small myocardial territory and other than representing a very uncommon coronary anomaly likely confers minimal clinical relevance. 

Anomalous LMCA arising from the right sinus of Valsalva is a rare coronary anomaly found in 0.0024% to 0.03% of the population [2, 4]. Depending on the anatomic relationship of the anomalous coronary artery to the aorta and the pulmonary trunk, the anomaly can be classified into four common courses: the posterior or retroaortic, the interarterial or preaortic, the septal or subpulmonary, and the pre-pulmonic or anterior course [8, 9]. The interarterial course has been associated with sudden cardiac death, particularly during exercise in athletes [10–13]. Surgical correction is indicated if there is evidence of myocardial ischemia or a history of syncope [14]. The prepulmonic, subpulmonary, and retroaortic courses are generally asymptomatic. 

 CTA can be used to characterize with great accuracy the course of the anomalous coronary artery, especially when differentiating between the subpulmonary or interarterial course [9]. These variations are important to be recognized prior to certain cardiac surgeries as failure to do so may result in unintentional transection of the aberrant blood vessel during the procedure. Accidental vascular compression of an anomalous LCXCA with prosthetic valvular rings has been reported during mitral and aortic valve replacements [15–17]. Informing the cardiothoracic surgeon along with defining the aberrant course may be lifesaving as special surgical considerations may be needed when performing such valvular procedures. 

In summary, we report a combination of two rare congenital coronary anomalies, a dual LADCA Type IV, and an anomalous right-sided LMCA with a subpulmonary course. This anomalous coronary anatomy is unlikely to be associated with sudden cardiac death or ischemia. Interventional cardiologists and cardiothoracic surgeons should be aware of these coronary variations to assure correct angiography interpretations and avoidance of complications. To the best of our knowledge, the combined presence of both coronary anomalies has not been previously described.
